# Eugenol accelerates intestinal stem cell regeneration to protect the intestinal barrier integrity through inhibiting JAK2/STAT3 signaling pathway in *Salmonella enteritidis*-challenged broiler chicks

**DOI:** 10.1186/s40104-025-01168-y

**Published:** 2025-03-10

**Authors:** Yaxue Lv, Nan Zeng, Yuqing Feng, Sheng Zhang, Xiaodan Zhou, Chunqi Gao

**Affiliations:** 1 College of Animal Science, Guangdong Provincial Key Laboratory of Animal Nutrition Control, Guangdong Laboratory for Lingnan Modern Agriculture, State Key Laboratory of Swine and Poultry Breeding Industry, South China Agricultural University, Guangzhou, 510642 China; 2 Guangdong Provincial Key Laboratory of Animal Nutrition Control, South China Agricultural University, Guangzhou, 510642 China

**Keywords:** Eugenol, Inflammatory response, Intestinal barrier, *Salmonella*, Stem cell activity

## Abstract

**Background:**

*Salmonella enteritidis* is a prevalent foodborne pathogen causing diseases in humans and poultry globally. While clove extract is known for its anti-inflammatory properties, its specific effects on gut injury and underlying mechanisms are not well understood.

**Methods:**

A total of 432 one-day-old male fast-growing yellow-feathered broilers with similar body weight were randomly assigned to 6 groups, the CON and S.E were fed a basal diet; the CE and S.E + CE received 300 mg/kg of clove extract in their diets; and the EUG and S.E + EUG had 180 mg/kg of eugenol added to their basal diets. Moreover, a newly established ex vivo culture model for chick intestinal organoids (IOs) was used to evaluate intestinal stem cell (ISC) activity.

**Results:**

*Salmonella enteritis* infection significantly reduced the growth performance and induced severe intestinal mucosa injury (*P* < 0.05). Dietary supplemented with clove extract or eugenol significantly improved average daily weight gain and feed intake, enhanced the structure and barrier function of the jejunum, reduced the bacterial load and diarrhea scores, promoted the proliferation and differentiation of ISCs, and diminished the efficiency, surface area, budding efficiency, and number of buds of intestinal organoids (*P* < 0.05). Both clove extract and eugenol down-regulated the protein expression of pro-inflammatory cytokines IL-1β, IL-6, and TNF-α. They also inhibited the excessive activation of the JAK2/STAT3 signaling pathway induced by *Salmonella enteritidis* infection in the jejunum tissues and crypts of chicks (*P* < 0.05).

**Conclusions:**

Eugenol, the active component in clove extract, alleviates intestinal inflammation by inhibiting the excessive activation of the JAK2/STAT3 signaling pathway. It promotes the proliferation and differentiation of ISCs, suppresses apoptosis, and accelerates ISCs-driven intestinal epithelial renewal in chicks, thereby maintaining the structural integrity and functional normalcy of the intestine.

**Supplementary Information:**

The online version contains supplementary material available at 10.1186/s40104-025-01168-y.

## Background

*Salmonella enteritidis* is a non-typhoidal pathogen that belongs to the intestinal subspecies of *Salmonella* (subspecies I). *Salmonella enteritidis* infection can stimulate a strong intestinal inflammatory response and modulate the destruction of tight junction proteins, thereby compromising the intestinal epithelial barrier. Additionally, by over activating the Wnt/β-catenin signaling pathway, it can increase the depth of intestinal crypts, leading to crypt hyperplasia and affecting the normal proliferation of intestinal stem cells [1, 2]. In our preliminary study, we observed that oral gavage with 1 × 10^10^ CFU of *Salmonella* significantly damages the tight junctions of chick intestinal epithelial cells. Furthermore, poultry products contaminated with this bacterium may serve as vehicles for transmission, posing a serious threat to human health. Due to its ability to infect various hosts and its high invasiveness, *Salmonella enteritidis* has become a major causative agent of human salmonellosis [3–5]. It may also trigger infections in mesenteric lymph nodes and various organs, such as the liver, spleen, ovaries, and gallbladder, potentially leading to systemic infections [6]. *Salmonella enteritidis* primarily invades the host by disrupting the intestinal barrier, increasing intestinal permeability, and allowing various harmful substances to enter the body, thus exacerbating the host’s inflammatory response.

Plant extracts possess growth-promoting and health-enhancing properties [7]. They interact with epithelial cells and gut microbiota to help maintain intestinal homeostasis. These extracts are primarily used to improve gut health in animals, including enhancing digestion, regulating secretions, and supporting intestinal tissue [8, 9]. In our previous study, we evaluated ten different plant extracts and found that clove extract exhibited the best inhibitory effect on *Salmonella enteritidis*. Clove extract is derived from the flower buds, leaves, and stems of clove, containing active components such as eugenol, acetyleugenol, and various ester compounds [10]. Among these, eugenol is the principal component, typically accounting for approximately 60%−80% of the total extract, depending on the extraction method [11]. Which exhibits significant antibacterial activity and has been shown to inhibit various pathogenic bacteria [12–14]. Previous study demonstrated that eugenol can significantly inhibit the mycelial growth and spore germination of *Fusarium graminearum*, disrupt the integrity and permeability of its cell membrane, and interfere with ergosterol synthesis on the cell membrane, thereby exhibiting antibacterial properties [14]. Furthermore, eugenol has shown synergistic inhibitory effects against foodborne *Salmonella *Typhi when combined with streptomycin and can suppress its biofilm formation [15]. Our GC–MS analysis revealed that the eugenol content in the clove extract we used is 60.01%, which is the main active component. Therefore, we hypothesize that the clove extract may primarily exert its antibacterial effects through the component eugenol. Although eugenol is recognized for its antibacterial and anti-inflammatory properties, its efficacy and mechanisms in alleviating *Salmonella enteritidis* infections remain to be elucidated.

The JAK2/STAT3 signaling pathway plays a crucial role in the regulation of physiological and pathological processes such as inflammation, immunity, and apoptosis [16–18]. Activation of JAK2 by inflammatory factors leads to the phosphorylation of its downstream protein STAT3, resulting in the overexpression of cytokine genes [19]. This includes excessive expression of inflammatory factors such as interleukin-6 (IL-6) and tumor necrosis factor-alpha (TNF-α), which can trigger severe inflammatory responses [20]. The JAK2/STAT3 signaling pathway mediates the inflammatory response following ischemia [21, 22] and its activation in macrophages induces an increase in HMGB1 expression and promotes the release of cytokines like TNF-α, thereby contributing to the inflammatory response [23]. Given its significant regulatory role, the JAK2/STAT3 signaling pathway presents new possibilities for further research into intestinal inflammation in poultry.

Therefore, the main objective of this study is to investigate the effects of *Salmonella enteritidis* on the intestinal stem cells of chicks, and to evaluate the role and mechanisms of clove extract and its primary active component, eugenol, in alleviating intestinal damage induced by *Salmonella enteritidis* infection.

## Materials and methods

### Animal ethics statement

Experimental animal procedures were conducted following the Guidelines for the Care and Use of Laboratory Animals established by South China Agricultural University (Guangzhou, China) and received approval from the Animal Ethics Committee of South China Agricultural University (Guangzhou, China).

### Reagents and instruments

The composition and nutritional components of the basal diet are shown in Table S1. The clove extract added to the diet was purchased from Beijing Zhongnong Limu Biotechnology Co., Ltd. (Beijing, China), and eugenol was purchased from Sigma-Aldrich (St. Louis, MO, USA) with a purity level greater than 98%. The reagents used in the study are listed in Table S2. Information on the software and instruments is provided in Table S3.

### Experimental design and treatments

**Fig. 1:**
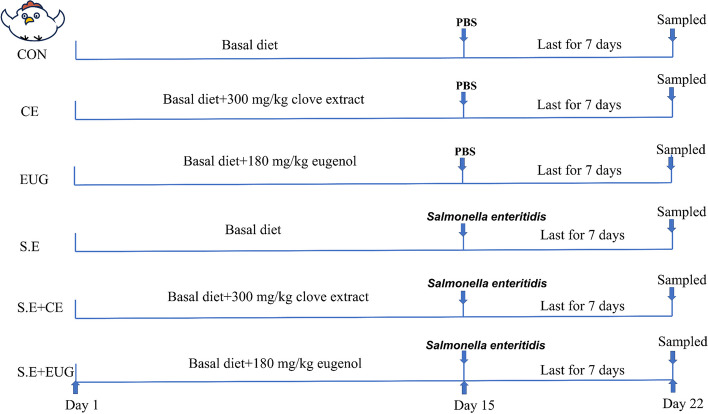
Schematic diagram of the experimental treatments

A total of 432 one-day-old male fast-growing yellow-feathered broilers with similar body weight were randomly assigned to 6 groups, each consisting of six replicates with 12 chicks per replicate. As shown in Fig. 1, the experimental groups were designed as follows: the CON and S.E were fed a basal diet; the CE and S.E + CE received 300 mg/kg of clove extract in their diets; and the EUG and S.E + EUG had 180 mg/kg of eugenol added to their basal diets. From d 1 to 21, all groups were provided with their designated diets. From d 15 to 21, the chicks in the 3 infection groups were subjected to daily gastric gavage with 1 mL PBS containing 1 × 10^10^ CFU of *Salmonella enteritis*, while the other three groups received the same volume of PBS as a control.

Throughout the experiment, data on feed intake, weight gain, and fecal consistency were collected. The average daily feed intake (ADFI), average daily gain (ADG), feed conversion ratio (FCR), and diarrhea scores were calculated. On d 22, 6 chicks from each replicate were randomly selected for blood collection via the wing vein, and measurements of small intestine length, weight, mucosal weight, and immune organ weight were recorded. The collected jejunal and serum samples were preserved for further analysis.

Diarrhea incidence was assessed using the following scoring criteria: 0 = normal feces, 1 = collapsed feces, 2 = unformed feces, and 3 = watery feces [24].

### Hematoxylin and eosin (H&E) staining

Fresh jejunal tissue was fixed in 4% paraformaldehyde for 48 h, followed by paraffin embedding. The tissue was sectioned into 5 μm thick slices and placed in a 37 °C oven overnight. Deparaffinization and hydration were performed according to the instructions of the H&E staining kit. The sections were stained with hematoxylin for 10 min and eosin for 2 min. After clearing with xylene, the slides were mounted with neutral resin. Under a microscope, cell nuclei appeared blue, while the cytoplasm showed pink or red. Image J software was utilized to measure villus height, crypt depth and the ratio of villus height to crypt depth.

### Measurement of intestinal permeability by *trans*-epithelial electrical resistance (TEER)

Preheat the instrument for 1 h and ensure correct connections between the electrodes and the power supply. The jejunal tissue to be tested is longitudinally opened along the mesentery and cut into blocks measuring 3 cm × 1 cm. These samples are placed in the two half-chambers of the Ussing Chamber, ensuring they are fully immersed in Krebs–Ringer buffer for stabilization. The entire setup is placed in a 37 °C water bath while being carbonated with a gas flow containing 95% O₂ and 5% CO₂. The transmembrane resistance value of the monolayer intestinal epithelial cells is recorded.

### Determination of *Salmonella* enrichment rate

The liver, spleen, breast muscle, and intestinal sample dilutions are prepared in serial dilutions. The 10⁻^1^, 10⁻^2^, 10⁻^3^, and 10⁻^4^ dilutions are transferred to their corresponding culture dishes, and xylose lysine deoxycholate (XLD) agar, pre-warmed in a 55 °C water bath, is added to each dish. The mixtures are thoroughly mixed to ensure homogeneity. After the agar solidifies, the plates are inverted and incubated at 37 °C for 16 to 24 h before counting the colonies.

### Transmission electron microscopy of the intestine

Fresh jejunal tissue is fixed in 2.5% glutaraldehyde for 48 h and cut to appropriate sizes. After rinsing with 0.1 mol/L phosphate buffer, the tissue is fixed in 1% osmium tetroxide and 0.1 mol/L phosphate buffer (pH 7.2). Following acid wash, dehydration, and embedding, the samples are double-stained with 3% uranyl acetate and lead citrate. Transmission electron microscopy (TEM) is used for observation and imaging.

### Western blotting

Jejunal tissue is homogenized with an appropriate amount of lysis buffer and protease inhibitors, followed by centrifugation to collect the supernatant. The protein concentration is measured using a BCA assay kit, and samples are adjusted to the same concentration. Sodium dodecyl sulfate polyacrylamide gel electrophoresis (SDS-PAGE) is performed to separate the protein samples and markers. The proteins are then transferred to a polyvinylidene fluoride (PVDF) membrane using a transfer apparatus under ice-cold conditions. After blocking with blocking buffer, the membrane is washed three times with Tris Buffered Saline with Tween-20 (TBST) and incubated sequentially with primary and secondary antibodies. Enhanced chemiluminescence (ECL) detection reagent is used, and the membrane is placed in a gel imaging system for automatic exposure to obtain bands of the target protein. Density values are analyzed using Image J software. The antibodies used are listed in Table S4.

### Immunofluorescence (IF) staining

Tissue sections undergo antigen retrieval using citrate buffer for 30 min, followed by permeabilization with Triton X-100 for 10 min. After blocking at room temperature for 10 min, the desired tissue area is circled with an immunohistochemistry pen, and the primary antibody is added and incubated overnight at 4 °C. The secondary antibody is then incubated at room temperature in the dark for 2 h. DAPI is used for staining, followed by a 10-min incubation at room temperature in the dark. Images are captured using a fluorescence inverted microscope, and fluorescence signal intensity is statistically analyzed with Image J software.

### Culture of chick intestinal organoids (IOs)

An approximately 10 cm segment of the intestine was taken and longitudinally cut open, followed by thorough washing with Dulbecco’s phosphate-buffered saline (DPBS). The intestinal segment was then incubated in DPBS containing 30 mmol/L disodium ethylenediaminetetraacetic acid (EDTA) and left on ice for 20 min to collect the crypt solution. Subsequently, the precipitate was washed 2 to 3 times with sterile complete medium (high DMEM/F12 containing 10% fetal bovine serum and 1% penicillin/streptomycin) and finally resuspended in 1 mL of complete medium. Crypts were counted under a microscope, setting the inoculation density to 40 to 50 crypts/well, approximately 20 μL/well. An equal volume of matrix gel was mixed and evenly dispensed into a 48-well plate, which was then placed in a 37 °C, 5% CO_2_ incubator for about 25 min until the matrix gel solidified completely. Each well received 250 μL of the prepared Wnt3a culture medium, with periodic imaging to document tissue growth.

Finally, organoid generation efficiency, budding efficiency, expansion area, and the number of buds were statistically analyzed to assess the activity of intestinal stem cells. Detailed components of the chicken crypt cell culture medium can be found in Table S5.

### Statistical analysis

To compare the differences between groups, one-way analysis of variance (ANOVA) is performed using IBM SPSS Statistics software (version 27.0). After confirming significant differences between groups through ANOVA, Duncan’s multiple range test was conducted for further analysis. The statistical results were expressed as mean ± SEM, with *P* < 0.05 considered statistically significant. This approach ensures an accurate analysis of data across groups and provides reliable statistical support to validate the experimental hypotheses.

## Results

### Eugenol improved the growth performance, decreased the diarrhea and bacterial load of chicks challenged with *Salmonella enteritidis*

As shown in Table 1, during 1–21 d, the ADFI and ADG of chicks in the CE and EUG groups were significantly higher than those in the CON group. Meanwhile, *Salmonella enteritidis* infection led to a notable reduction in the average daily gain and feed intake of the chicks. Feeding with clove extract and eugenol significantly improved the daily gain and feed intake of infected chicks. Additionally, the inclusion of eugenol significantly lowered the feed-to-gain ratio. Feeding clove extract and eugenol significantly reduced the diarrhea scores in infected chicks. Compared to the CON, *Salmonella* infection significantly elevated the bacterial load of *Salmonella* in the liver, spleen, muscle, and various intestinal segments of yellow feather broilers (Table 2). The addition of clove extract and eugenol significantly diminished the bacterial load.

**Table 1:** Effect of dietary supplemental with eugenol on growth performance in chicks challenged with *Salmonella enteritidis*^1^

Item	Treatments						SEM	*P*-value
	**CON**	**CE**	**EUG**	**S.E**	**S.E + CE**	**S.E + EUG**		
Average daily gain, g	17.22^b^	18.13^a^	18.49^a^	16.30^c^	17.22^b^	17.32^b^	0.13	< 0.001
Average daily feed intake, g	32.82^b^	34.56^a^	33.96^a^	30.87^c^	32.15^b^	31.89^b^	0.24	< 0.001
Feed to gain ratio	1.91	1.91	1.84	1.90	1.87	1.84	0.01	0.146
Diarrhea score	1.08b^c^	0.50^c^	0.50^c^	2.67^a^	1.58^b^	1.67^b^	0.16	< 0.001

^1^*CON* Control group, *CE* Clove extract group*, EUG* Eugenol group*, S.E Salmonella enteritis* group*, S.E* + *CE Salmonella enteritidis* treated with clove extract group*, S.E* + *EUG Salmonella enteritidis* treated with eugenol group, *SEM* Standard error of the mean

^a^^−^^c^Means within a row with different superscripts are significantly different (*P* < 0.05)

**Table 2:** Effect of dietary supplemental with eugenol on bacterial load in viscera and small intestine of chicks challenged with *Salmonella enteritidis*^1^

Item	Treatments						SEM	*P*-value
	**CON**	**CE**	**EUG**	**S.E**	**S.E + CE**	**S.E + EUG**		
Liver, log_10_ CFU/g	0.00^c^	0.00^c^	0.00^c^	2.45^a^	1.68^b^	1.73^b^	0.14	< 0.001
Spleen, log_10_ CFU/g	0.00^c^	0.00^c^	0.00^c^	2.68^a^	2.02^b^	2.02^b^	0.21	< 0.001
Muscle, log_10_ CFU/g	0.00^c^	0.00^c^	0.00^c^	2.23^a^	1.34^b^	1.15b^c^	0.07	< 0.001
Duodenum, log_10_ CFU/g	0.00^b^	0.00^b^	0.00^b^	3.18^a^	2.19^b^	2.26^b^	0.33	< 0.001
Jejunum, log_10_ CFU/g	0.00^b^	0.00^b^	0.00^b^	3.52^a^	2.59^b^	2.58^b^	0.39	< 0.001
Ileum, log_10_ CFU/g	0.00^b^	0.00^b^	0.00^b^	3.79^a^	2.93^b^	3.01^b^	0.44	< 0.001

^1^*CON* Control group, *CE* Clove extract group*, EUG* Eugenol group*, S.E Salmonella enteritis* group*, S.E* + *CE Salmonella enteritidis* treated with clove extract group*, S.E* + *EUG Salmonella enteritidis* treated with eugenol group, *SEM* Standard error of the mean

^a^^−^^c^Means within a row with different superscripts are significantly different (*P* < 0.05)

### Eugenol ameliorates *Salmonella enteritidis*-induced intestinal injury

**Fig. 2:**
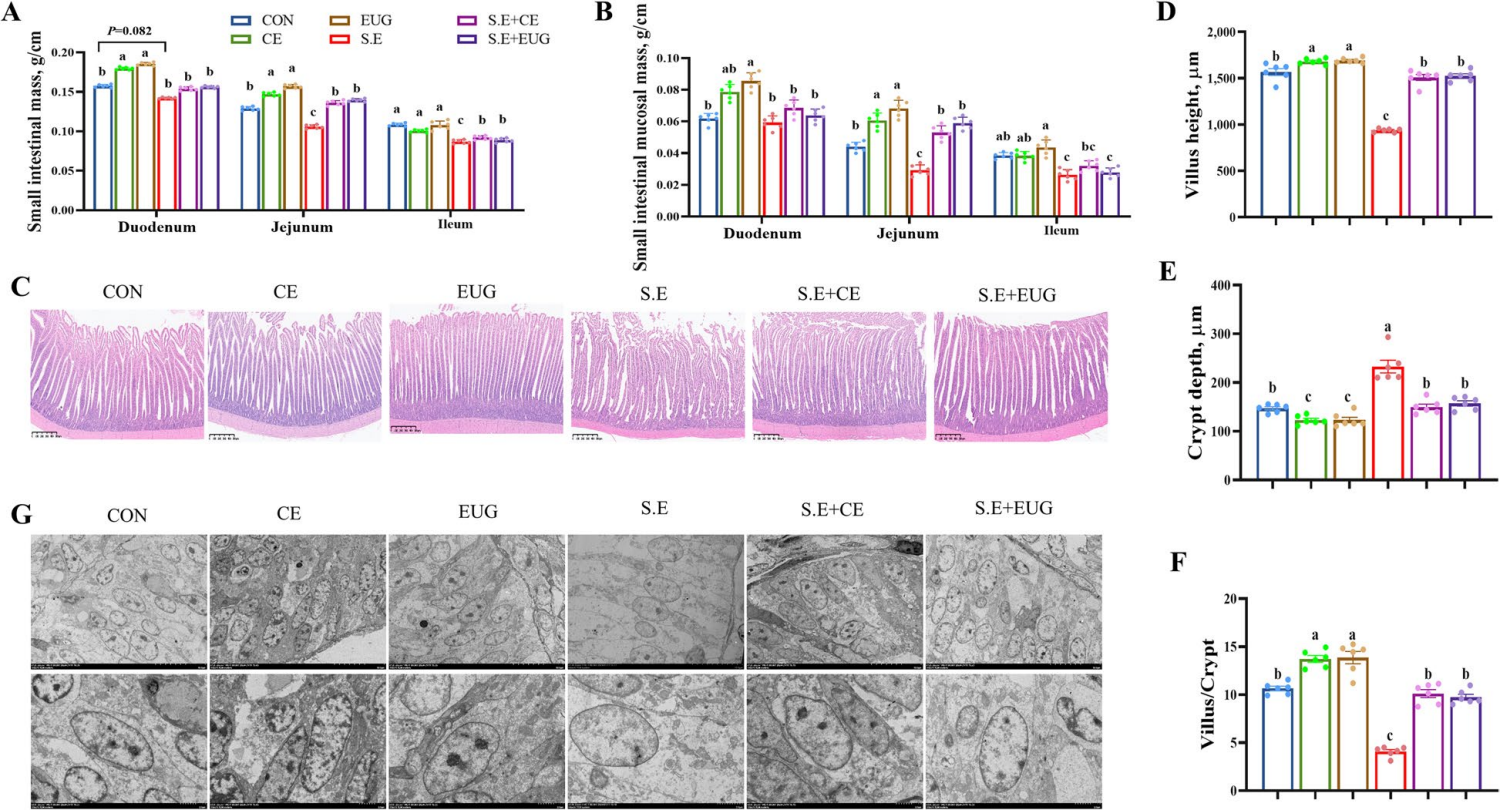
Eugenol ameliorates *Salmonella enteritidis*-induced intestinal injury. **A** Small intestine weight per unit length. **B** Weight of small intestinal mucosa per unit length. **C** Images stained with hematoxylin and eosin (100 × magnification, scale bar = 200 μm). **D** Villus height. **E** Crypt depth. **F** Villus height/crypt depth. **G** Crypt structure of the jejunum under a scanning electron microscope (magnification 250 ×). Data are shown as mean ± SEM (*n* = 6). Differences between groups were indicated by different lowercase letters (*P* < 0.05)

In comparison to the CON, the addition of clove extract and eugenol significantly elevated the duodenal and jejunal weight per unit length and mucosal weight in yellow-feathered broilers, with a more pronounced effect on the jejunum and no significant impact on the ileum. *Salmonella* infection significantly reduced the jejunal weight and mucosal weight in yellow-feathered broilers, while the addition of clove extract and eugenol improved the unit weight and mucosal weight of the jejunum and ileum in infected chicks and alleviated intestinal lesions (Fig. 2A and B, *P* < 0.05). Compared to the CON, the diets supplemented with clove extract and eugenol significantly heightened the villus height and villus-to-crypt ratio in the jejunal tissue of yellow-feathered broilers, while diminishing crypt depth; *Salmonella* infection significantly reduced villus height and villus-to-crypt ratio and shallowed crypt depth. The addition of clove extract and eugenol effectively heightened the villus height and villus-to-crypt ratio in infected chicks and reduced crypt depth (Fig. 2C–F, *P* < 0.05). Scanning electron microscopy observations revealed that in the *Salmonella*-infected group, the crypt cells of the small intestine were enlarged, and the number of cells was reduced, while the addition of clove extract and eugenol alleviated this phenomenon (Fig. 2G).

### Eugenol alleviates *Salmonella enteritidis*-induced intestinal epithelial barrier dysfunction

**Fig. 3:**
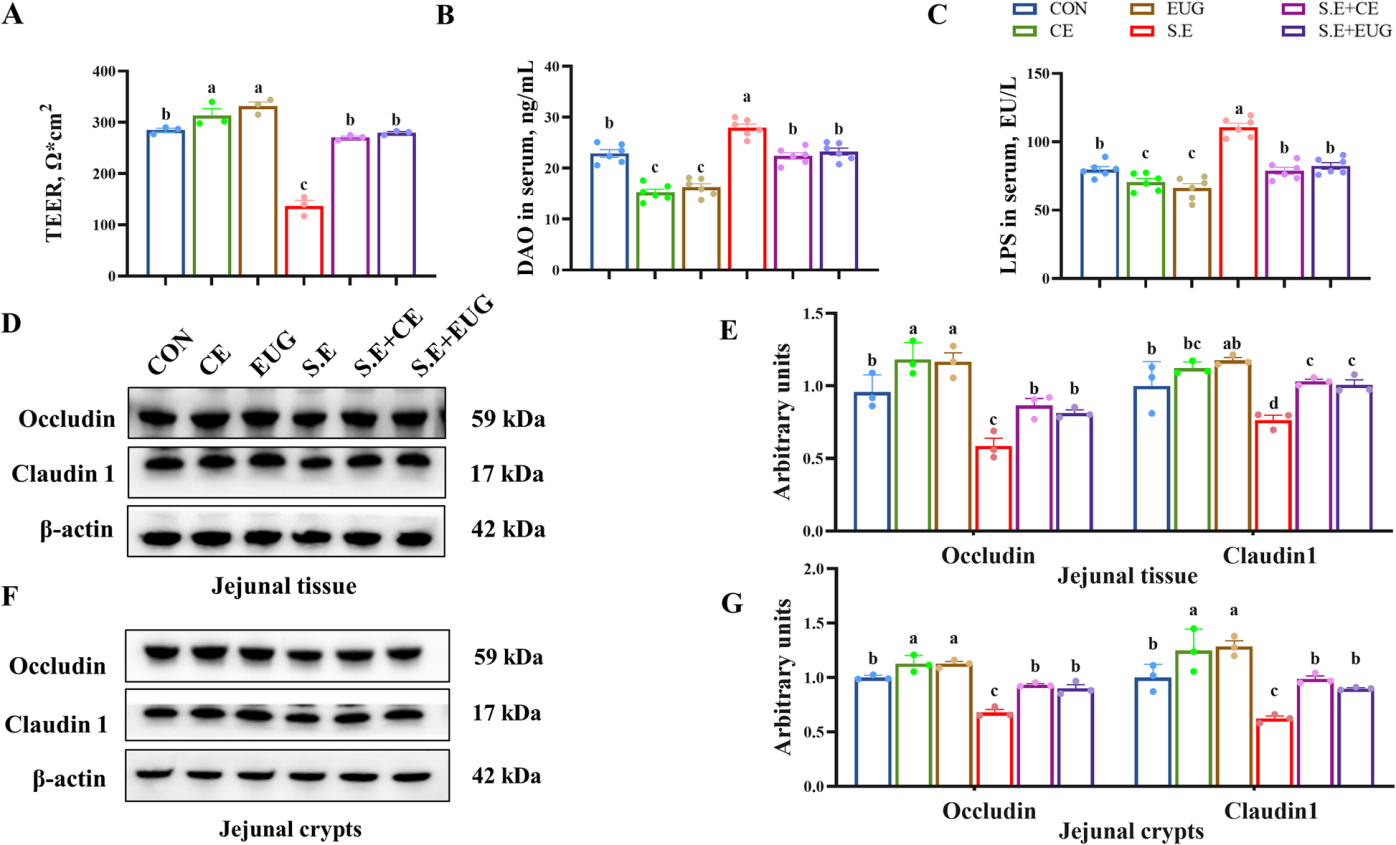
Eugenol alleviates *Salmonella enteritidis*-induced intestinal epithelial barrier dysfunction. **A** Jejunal transepithelial electrical resistance (TEER). **B** Serum diamine oxidase (DAO) activity. **C** Serum LPS content. **D** and **E** Jejunal tissue protein levels of Occludin and Claudin-1. **F** and **G** Jejunal crypt protein levels of Occludin and Claudin 1. Data are shown as mean ± SEM (*n* = 3). Different lowercase letters indicated significant differences between groups (*P* < 0.05)

Compared to the CON, the addition of clove extract and eugenol to the diet significantly promoted the levels of tight junction proteins Occludin and Claudin 1 in the jejunal tissue and crypts and elevated the TEER values (Fig. 3D–G, *P* < 0.05). Additionally, it reduced the activity of serum diamine oxidase (DAO) and levels of lipopolysaccharides (LPS), showing significant effects on the infected group as well (Fig. 3B and C, *P* < 0.05).

### Eugenol alleviates *Salmonella enteritidis*-induced impairment of ISC proliferation and differentiation

**Fig. 4:**
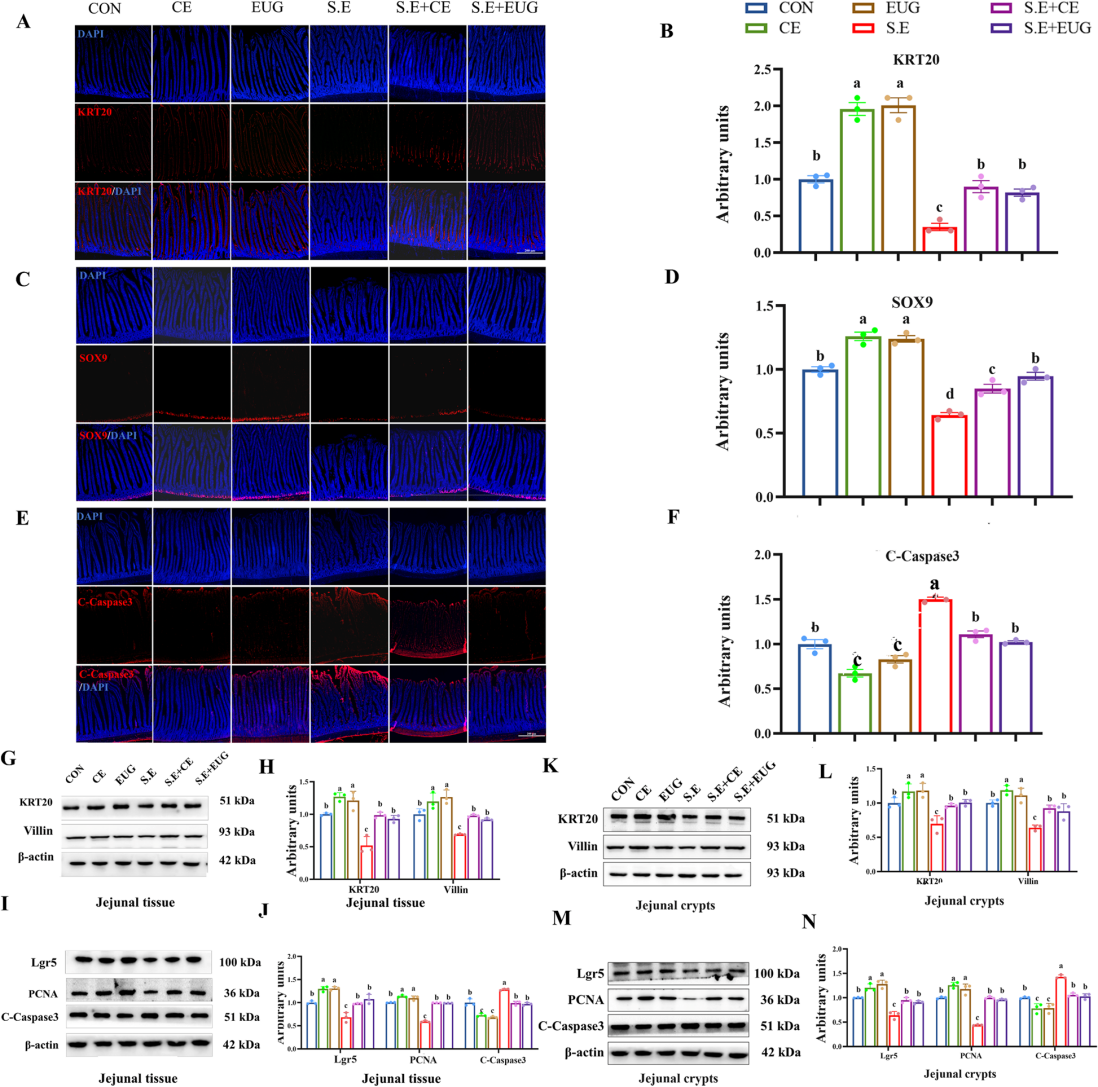
Eugenol alleviates *Salmonella enteritidis*-induced impairment of ISCs proliferation and differentiation. **A**–**F** Jejunal IF staining of keratin 20 (KRT20), SOX9, and C-caspase-3 (100 × magnification, scale bar = 200 μm). **G **and **H** Jejunal tissue protein levels of KRT20 and Villin. **I** and **J** Jejunal tissue protein levels of Lgr5, PCNA, C-caspase-3. **K** and **L** Jejunal crypt protein levels of KRT20 and Villin. **M** and **N** Jejunal crypt protein levels of Lgr5, PCNA, C-caspase-3. Data are shown as mean ± SEM (*n* = 3). Differences between groups were indicated by different lowercase letters (*P* < 0.05)

Compared to the CON, the administration of clove extract and eugenol significantly enhanced the IF signal intensity of KRT20 (Fig. 4A and B, *P* < 0.05). Furthermore, these treatments elevated the number of the secretory progenitor marker SOX9 and reduced the IF signal intensity of cleaved caspase-3 (C-caspase-3) (Fig. 4C–F, *P* < 0.05). There was also a notable increase in the protein levels of terminal differentiation marker KRT20 and absorptive cell marker Villin (Fig. 4G–L, *P* < 0.05). Additionally, the protein levels of ISC marker Lgr5 and proliferative cell marker proliferating cell nuclear antigen (PCNA) were significantly elevated, while the apoptotic marker C-caspase-3 protein level was markedly reduced (Fig. 4I–N, *P* < 0.05). Notably, these treatments also significantly alleviated the damage caused by *Salmonella* infection, which is associated with intestinal inflammation.

### Eugenol ameliorates *Salmonella enteritidis*-induced intestinal injure through activating ISC expansion

**Fig. 5:**
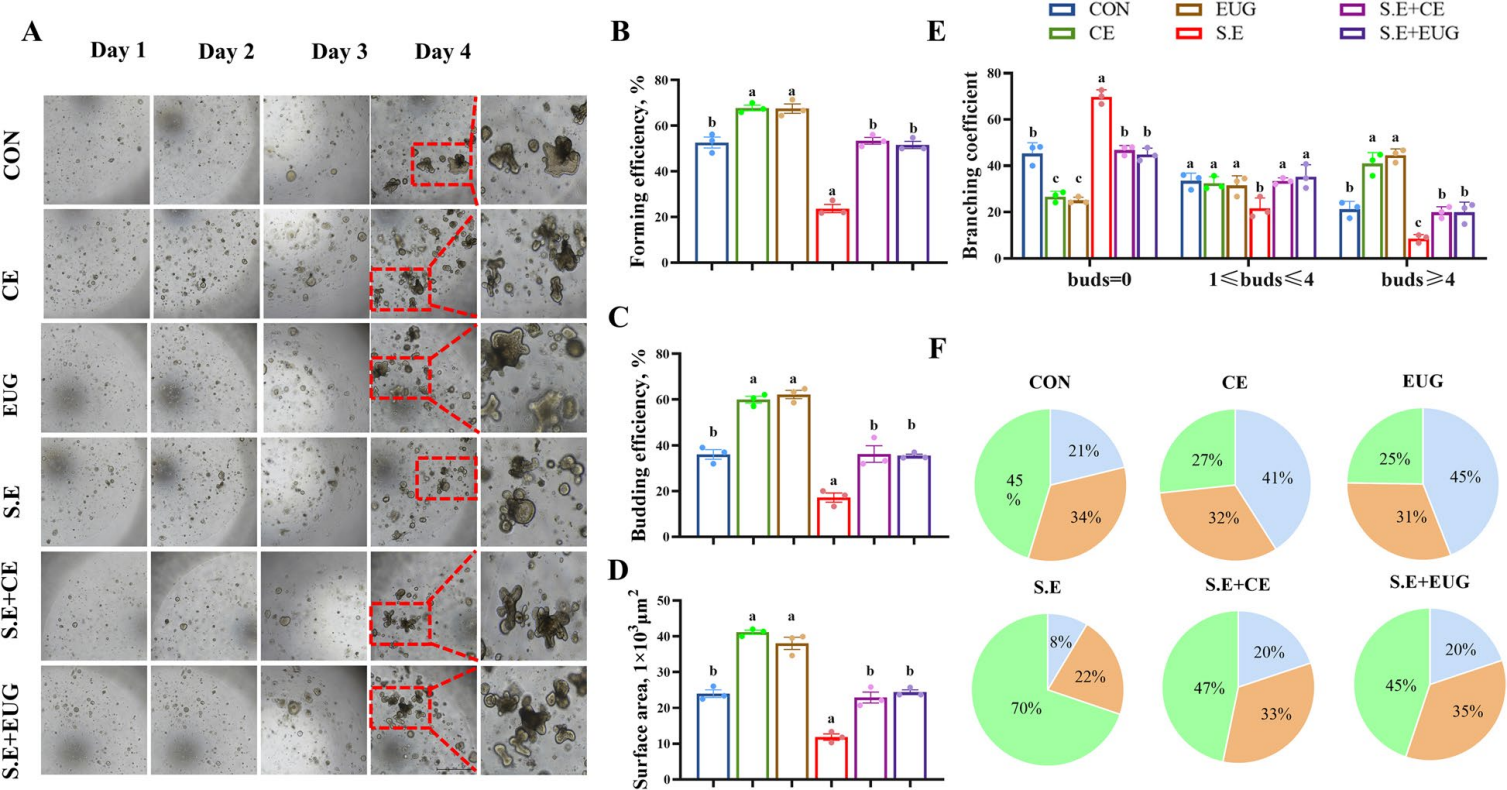
Eugenol ameliorates *Salmonella enteritidis*-induced intestinal injure through activating ISCs expansion. **A** Jejunal IO images of different groups on d 1, d 2, and d 4 (magnification of 40 × , scale bar = 200 μm). **B** and **C **IO formation efficiency (**B**) and budding efficiency (**C**) at d 4. **D** IO surface area on d 1, 2, and 4. **E** IO budding numbers were measured on d 4. **F** Branching coefficient on d 4 of IO. Data are shown as mean ± SEM (*n* = 3). Different lowercase letters indicated significant differences between groups (*P* < 0.05)

Compared to the CON, the clove extract and eugenol groups exhibited a significant increase in intestinal organoid generation efficiency, budding efficiency, and surface area; however, *Salmonella*-induced enteritis significantly inhibited organoid activity, resulting in a distinct cystic appearance (Fig. 5A–D, *P* < 0.05). The addition of clove extract and eugenol rescued the growth deficiencies induced by *Salmonella enteritis*, leading to a notable increase in the number of buds. These results indicate that eugenol promotes the in vitro expansion of ISCs, thereby accelerating intestinal epithelial regeneration (Fig. 5E and F, *P* < 0.05).

### Eugenol reduces inflammation levels and inhibits the overactivation of the JAK2/STAT3 signaling pathway induced by *Salmonella enteritidis*

**Fig. 6:**
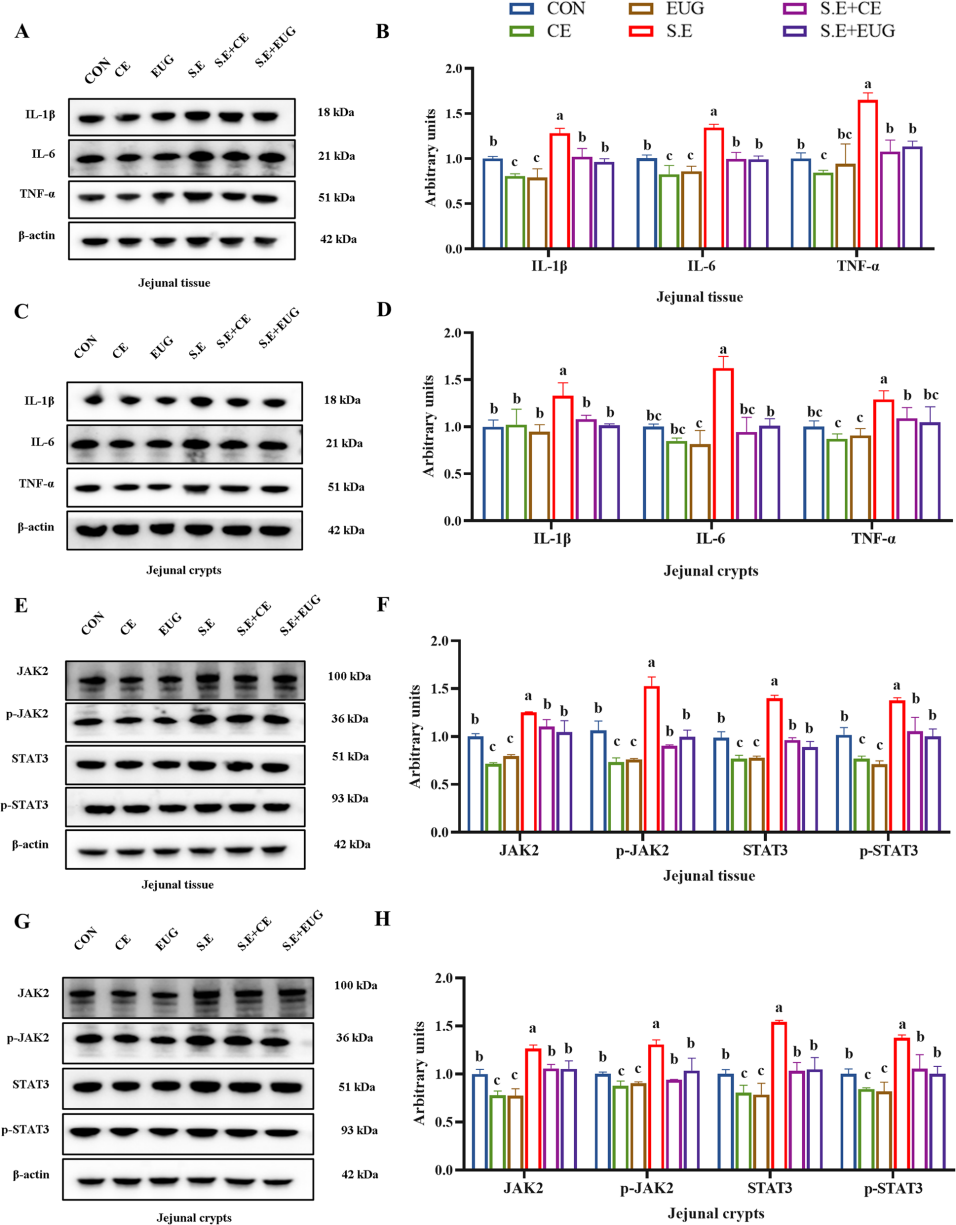
Eugenol reduces inflammation levels and inhibits the overactivation of the JAK2/STAT3 signaling pathway induced by *Salmonella enteritidis*. **A**–**D** Inflammatory factor-related protein levels in jejunal tissue and jejunal crypt. **E**–**H** JAK2/STAT3 signaling pathway-related protein levels in jejunal tissue and jejunal crypt. Data are shown as mean ± SEM (*n* = 3). Different lowercase letters indicated significant differences between groups (*P* < 0.05)

Compared to the CON, clove extract and eugenol notably reduced the expression levels of pro-inflammatory cytokines IL-1β, IL-6, and TNF-α in the jejunal tissue and crypts of yellow feather broilers; *Salmonella* infection significantly elevated the levels of these inflammatory factors. The addition of clove extract and eugenol significantly lowered the inflammation levels in the infected chicks (Fig. 6A–D, *P* < 0.05). Furthermore, both clove extract and eugenol significantly reduced the protein expression levels of JAK2, p-JAK2, STAT3, and p-STAT3 in the jejunal tissues and crypts of yellow feather broilers. *Salmonella* infection led to the overactivation of the JAK2/STAT3 signaling pathway. The addition of clove extract and eugenol effectively inhibited the excessive activation of the JAK2/STAT3 signaling pathway induced by *Salmonella* infection (Fig. 6E–H, *P* < 0.05).

### Inhibition of JAK2/STAT3 signaling is essential for the resistance of eugenol against inflammatory responses induced by* Salmonella enteritidis *in ex vivo model

Crypts were isolated from the jejunal tissues of each group of chicks and cultured in vitro. The addition of clove extract and eugenol elevated the protein expression levels of Occludin, Claudin 1, Lgr5, PCNA, KRT20, and Villin in the crypts of *Salmonella*-infected chicks, while reducing the levels of C-Caspase3, IL-1β, IL-6, and TNF-α. Moreover, it downregulated the expression of JAK2, p-JAK2, STAT3, and p-STAT3 proteins. The addition of clove extract or an equivalent amount of its active component, eugenol, alleviated intestinal inflammation by inhibiting the excessive activation of the JAK2/STAT3 signaling pathway. This, in turn, restored the proliferation capacity and activity of ISCs, maintaining the structural integrity and normal function of the intestine.

## Discussion

The addition of plant extracts in feed can promote animal growth and maintain gut health. Research has shown that plant extracts can enhance digestive enzyme activity and improve feed palatability, thereby boosting animal growth performance [7, 25, 26]. Clove can enhance the activity of digestive enzymes in the gut, alter the texture of feed in the intestines, reduce the viscosity of digesta, and improve the absorption efficiency of nutrients from feed, which promotes production performance. Eugenol, the main component of clove extract, varies in content from 60%–80% due to differences in species and processing techniques. Dietary supplemental with eugenol can improve the growth performance of broiler chickens, support gut health and immune function, and maintain meat quality without adverse effects [27]. In our study, we observed that the inclusion of 300 mg/kg clove extract and 180 mg/kg eugenol in the diet significantly elevated the average daily weight gain and feed intake of chicks while effectively reducing the incidence of diarrhea, and the effects between the two supplements were not significantly different.

A healthy gut microbiota and its metabolites play a protective role in maintaining intestinal barrier integrity, and the colonization resistance of the gut microbiota is a key mechanism for resisting pathogens [28–32]. *Salmonella* enhances its colonization ability by altering the composition of the gut microbiota, specifically by reducing Firmicutes and amplifying Bacteroidetes and Actinobacteria [33]. Studies have shown that plant extracts such as chlorogenic acid and Madagascar dragon's blood extract can reduce the concentration of *Salmonella* in animals [34, 35]. Previous studies have shown that eugenol can bolster the population of *Lactobacillus*, which is part of the Firmicutes phylum, in the chicken intestine, thereby preventing the growth and colonization of *Salmonella* and reducing their pathogenicity [36]. Our present findings indicated that the addition of clove extract and eugenol significantly lowered the prevalence of *Salmonella* in the liver, spleen, and intestines of infected chicks, demonstrating their effectiveness in reducing *Salmonella* infections.

The structure and function of the intestine are important indicators for assessing health status [37]. *Salmonella* infection causes damage to intestinal structures, characterized by reduced villus height and elevated crypt depth, which in turn affects nutrient absorption. Plant extracts have been shown to improve the morphology of the intestinal mucosa and promote the secretion of digestive enzymes, thereby enhancing intestinal absorption capacity [38, 39]. Our study similarly demonstrated that the addition of clove extract and eugenol significantly reduced the severity of intestinal lesions, improving villus height and villus-to-crypt ratio [40].

The intestinal epithelial barrier is a crucial structure for preventing pathogen invasion, with tight junctions playing a vital role in maintaining barrier function [41, 42]. Research has indicated that enteric pathogens compromise intestinal barriers by disrupting tight junction structures [43]. Many intestinal pathogenic bacteria disrupt tight junctions structures by altering the cytoskeleton or affecting specific proteins of tight junctions, such as Occludin, Claudin 1, and ZO1, leading to diarrhea [44, 45].In our experiments, *Salmonella*-infected chicks exhibited diminished TEER values and reduced expression of tight junction proteins Occludin and Claudin 1. However, the addition of clove extract and eugenol significantly elevated TEER values and enhanced the expression of tight junction proteins. These findings are consistent with previous studies demonstrating the essential role of clove extract and eugenol in maintaining the integrity of the intestinal barrier structure [46, 47].

ISCs play a crucial role in maintaining intestinal homeostasis through self-renewal and differentiation to repair damaged intestinal mucosa [48–50]. The mechanisms by which *Salmonella* infection regulates ISCs remain inconclusive. Some studies have found that *Salmonella* infection augments the number of Paneth cells and intestinal epithelial cells, accelerating the excessive proliferation of ISCs [51]. In contrast, other studies suggest that *Salmonella* may inhibit the expression of Lgr5, leading to damage in intestinal epithelial cells and enhanced apoptosis [52]. Our study demonstrated that enteritis-causing *Salmonella* infection resulted in diminished ISCs activity, as evidenced by the downregulation of Lgr5, PCNA, KRT20, and Villin expression. These findings are consistent with previous results indicating that the *Salmonella* strain used in this experiment inhibits the proliferation and differentiation of stem cells [53]. This discrepancy may arise from differences in the virulence genes of various *Salmonella* serotypes. Furthermore, plant extracts have been shown to maintain stem cell activity and regulate their differentiation fate [54, 55]. The addition of clove extract and eugenol significantly enhanced the expression of stem cell marker proteins, supporting their role in promoting cell proliferation and differentiation.

**Fig. 7:**
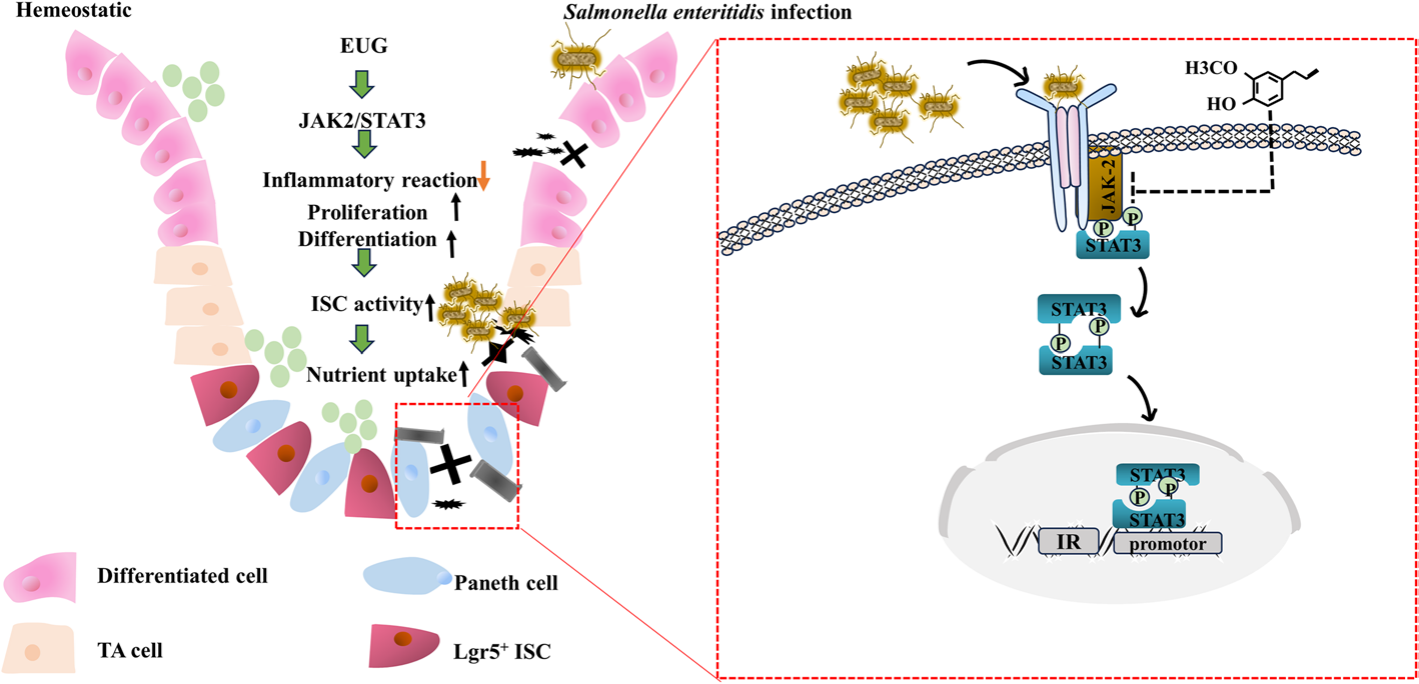
Eugenol promotes the proliferation and differentiation of ISCs by inhibiting the excessive activation of the JAK2/STAT3 signaling pathway, while also suppressing apoptosis, thereby accelerating ISCs-driven intestinal epithelial renewal

Under *Salmonella* infection, intestinal sensitivity to inflammation significantly increases, activating inflammatory response pathways and inducing the production of cytokines such as LITAF and IFN-γ [56, 57].Clove extract is widely used in the healthcare field, and its anti-inflammatory effects are well recognized [58, 59] .Numerous studies have demonstrated that eugenol exhibits strong anti-inflammatory capabilities, effectively inhibiting leukocyte migration. The study demonstrated that it reduced the expression of mRNA for *TNF-α*, *IL-1β*, *IL-2*, and *IL-18*, thereby alleviating the inflammatory response induced by *Salmonella* Typhimurium in chicks [60, 61]. Our study found that clove extract and eugenol effectively downregulated the expression of inflammatory factors such as TNF-α, IL-1β, and IL-6, thereby reducing intestinal inflammation. The JAK/STAT signaling pathway is regarded as a critical communication node for cellular functions and is evolutionarily conserved. This pathway consists of ligand-receptor complexes, Janus kinases, and signal transducers and activators of transcription [62]. It reveals significant differences in JAK/STAT signal transduction within the intestinal mucosa between two genetically distinct chicken lines. A Research indicates that the JAK2/STAT3 signaling pathway plays a pivotal role in regulating various inflammatory and anti-inflammatory pathways [63]. This pathway is capable of mediating inflammatory responses following ischemia and is involved in the regulation of the release of cytokines such as TNF-α in macrophages. Furthermore, the IL-6-mediated JAK2/STAT3 signaling pathway has been demonstrated to possess anti-inflammatory effects in inflammation and oxidative stress [63–65]. Adding *Lactobacillus plantarum* DPP8 and *Lactobacillus acidophilus* C7282 to the diet can block the JAK/STAT signaling pathway, thereby reducing intestinal inflammation induced by *Salmonella* infection [66]. These findings underscore the important regulatory role of the JAK2/STAT3 signaling pathway in inflammation (Fig. 7).

## Conclusion

Eugenol alleviates intestinal inflammation by inhibiting the excessive activation of the JAK2/STAT3 signaling pathway. It promotes the proliferation and differentiation of ISCs, suppresses apoptosis, and accelerates ISCs-driven intestinal epithelial renewal in chicks, thereby maintaining the structural integrity and functional normalcy of the intestine.

## Supplementary information

Additional file 1: Table S1 The ingredients and nutrient composition of the basal diet. Table S2 Reagents used in this study. Table S3 Instruments and software used in this study. Table S4 Antibodies used in this study. Table S5 Chick crypt cell medium composition.

## Data Availability

If reasonably necessary, data explaining the results of this study can be obtained from the corresponding author.

## Abbreviations

ADFI: Average daily feed intake
ADG: Average daily gain
BCA: Bicinchoninic acid
C-caspase-3: Cleaved caspase-3
DAO: Diamine oxidase
DAPI: 4′,6-Diamidino-2-phenylindole
DPBS: Dulbecco’s phosphate-buffered saline
FBS: Fetal bovine serum
H&E: Hematoxylin and eosin
IF: Immunofluorescence
IL-1β: Interleukin-1β
IL-6: Interleukin-6
IO: Intestinal organoid
ISC: Intestinal stem cell
KRT20: Keratin 20
PBS: Phosphate-buffered solution
PCNA: Proliferating cell nuclear antigen
p-JAK2: Phosphorylated Janus kinase 2
p-STAT3: Phosphorylated signal transducer and activator of transcription 3
PVDF: Polyvinylidene fluoride
SB202190: 4-(4-Fluorophenyl)-2-(4-hydroxyphenyl)-5-(4-pyridyl)1H-imidazole
TEER: Trans‐epithelial electrical resistance
TJ: Tight junction
TNF-α: Tumor necrosis factor-α
