# Supplementary material for: Eugenol accelerates intestinal stem cell regeneration to protect the intestinal barrier integrity through inhibiting JAK2/STAT3 signaling pathway in Salmonella enteritidis-challenged broiler chicks
Source: J Anim Sci Biotechnol. 2025 Mar 10;16:40. doi: 10.1186/s40104-025-01168-y (PMC11892138; doi:10.1186/s40104-025-01168-y)
Supplement: Supplementary file 1 — Additional file 1: Table S1 The ingredients and nutrient composition of the basal diet. Table S2 Reagents used in this study. Table S3 Instruments and software used in this study. Table S4 Antibodies used in this study. Table S5 Chick crypt cell medium composition. [file 40104_2025_1168_MOESM1_ESM.docx]

Supporting Information

**Table S1.** The ingredients and nutrient composition of the basal diet **Table S2.** Reagents used in this study.

**Table S3.** Instruments and software used in this study.

**Table S4.** Antibodies used in this study.

**Table S5.** Chick crypt cell medium composition.

# Supplementary Table

# Table S1. Ingredients and nutrient composition of the basal diet (air-dry basis)

| Item | Content |
| --- | --- |
| Ingredients, % as fed |  |
| Corn | 61.00 |
| Soybean meal | 30.00 |
| Fish meal | 2.70 |
| Soybean oil | 2.20 |
| DL-methionine (98%) | 0.30 |
| Calcium hydrogen phosphate | 1.50 |
| Limestone | 1.00 |
| Sodium chloride | 0.30 |
| Vitamin and mineral premix^1^ | 1.00 |
| Total | 100.00 |
| Calculated values of nutrient levels, %^2^ |  |
| Metabolizable energy, MJ/kg | 12.12 |
| Crude protein | 19.90 |
| Lysine | 1.15 |
| Methionine | 0.60 |
| Methionine + Cystine | 0.94 |
| Calcium | 1.00 |
| Available phosphorus | 0.47 |

^1^Supplied per kilogram of diet: vitamin A 12 000 IU, vitamin D_3_ 3 000 IU, vitamin E 20 mg, vitamin K_3_ 3.5 mg, vitamin B_1_4.0 mg, vitamin B_2_ 10.0 mg, vitamin B_6_ 8.0 mg, vitamin B_12_ 0.05 mg, D-pantothenic acid 25 mg, nicotinic acid 60 mg, biotin 0.2 mg, folic acid 2.5 mg, Fe (as ferrous sulfate) 90 mg, Cu (as copper sulfate) 30 mg, iodine (as potassium iodide) 0.8 mg, Zn (as zinc sulfate) 100 mg, Mn (as manganese sulfate) 110 mg, Se (as sodium selenite) 0.10 mg.

^2^Metabolizable energy was a calculated value, while the others were measured values.

# Table S2. Reagents used in this study

| Reagent/Material | Source |
| --- | --- |
| Advanced DMEM/F1 | Gibco |
| B27 supplemen | Invitrogen |
| Chick | Guangdong Baiyun District Xinwang Poultry Industry Co., Ltd. |
| Clove extract | Delacon Biotechnik GmbH |
| Diamine oxidase | Shanghai enzyme-linked biotechnology co., Ltd |
| DMEM | Shenzhen Maikos Biotechnology Co., Ltd |
| Eugenol (≥ 98%) | Sigma-Aldrich |
| Fast Transfer | Changzhou Boyi Biotechnology Co., Ltd |
| FBS | Gibco |
| FuturePAGE ™ 4-12% Protein Prefabricated Glue | Changzhou Boyi Biotechnology Co., Ltd |
| Hematoxylin eosin (HE) staining kit | Biyuntian Biotechnology Company Limited |
| Lipopolysaccharides | Shanghai enzyme-linked biotechnology co., Ltd |
| N2 supplement | Invitrogen |
| PH7.0 Sodium Chloride-Peptone Buffer | Guangdong Huankai Microbial Technology Co., Ltd |
| Pre-stained protein Marker II (10-200) | Wuhan Saiweier Biotechnology Co., Ltd |
| Rapid protein-free blocking solution | Changzhou Boyi Biotechnology Co., Ltd |
| Reconstitution Buffer 2 (BSA) | R&D System |
| RIPA Lysate | Beijing Baitek Biotechnology Co., Ltd |
| RSPO1 | R&D System |
| SB202190 | Sigma-Aldrich |
| Sterile homogenization bag | Guangdong Huankai Microbial Technology Co., Ltd |
| TBST buffer (10 ×, PH = 7.4) | Changzhou Boyi Biotechnology Co., Ltd |
| Tryptic soybean | Guangdong Huankai Microbial Technology Co., Ltd |
| Ultra-sensitive ECL chemiluminescence kit | Suzhou Xinsaimei Biotechnology Co., Ltd |
| Xylose lysine deoxybile salt agar | Qingdao Haibo Biotechnology Co., Ltd |

# Table S3. Instruments and software used in this study

| Instruments and Software | Source |
| --- | --- |
| Microscope | Nikon |
| NIS-Elements Viewer software | Nikon |
| Scanning electron microscope | Carl Zeiss |
| Semiautomatic Paraffin Slicer | Thermo Fisher Scientific |
| SPSS analysis software | SPSS Inc |
| Stratagene MxPro 3005Pthermocycler | Santa Clara |

# Table S4. Antibodies used in this study

| Antibody | Type | Source | Product Number |
| --- | --- | --- | --- |
| C-caspase-3 | Rabbit | Cell Signaling Technology | #9664 |
| Claudin‐1 | Rabbit | Zen BioScience | # 680135 |
| IL-1β | Rabbit | ABclonal Technology | #A23484 |
| IL-6 | Rabbit | ABclonal Technology | # A11115 |
| JAK2 | Mouse | Zen BioScience | #R24775 |
| KRT20 | Rabbit | Cell Signaling Technology | #13063 |
| Lgr5 | Rabbit | Zen BioScience | #380973 |
| Occludin | Rabbit | Zen BioScience | #502601 |
| PCNA | Mouse | Cell Signaling Technology | #2586 |
| p-JAK2 | Rabbit | ABclonal Technology | #AP0373 |
| p-STAT3 | Rabbit | ABclonal Technology | #AP0715 |
| SOX9 | Rabbit | Cell Signaling Technology | #82630 |
| STAT3 | Rabbit | ABclonal Technology | #A22434 |
| TNF-α | Rabbit | WANLEIBIO | #WL01581 |
| Villin | Mouse | Santa Cruz Biotechnology | #SC-58897 |
| β-actin | Mouse | Santa Cruz Biotechnology | #SC-130065 |

# Table S5. Chick crypt cell medium composition

| Item | Working solution | Source |
| --- | --- | --- |
| Advanced DMEM/F12 | 45% | Gibco |
| B27 | 1× | Invitrogen |
| EGF | 50 ng/mL | PeproTech |
| FBS | 10% | Gibco |
| Glutamine | 1× | Sigma Aldrich |
| N2 | 1× | Invitrogen |
| N-acetyl-cysteine | 1 mmol/L | Sigma Aldrich |
| Noggin | 100 ng/mL | PeproTech |
| R-Spondin1 | 500 ng/mL | R&D |
| SB202190 | 10 μmol/L | Sigma Aldrich |
| Wnt3a-CM | 45% |  |
